# Supplementary material for: Child public health indicators for fragile, conflict-affected, and vulnerable settings: A scoping review
Source: PLOS Glob Public Health. 2025 Mar 14;5(3):e0003843. doi: 10.1371/journal.pgph.0003843 (PMC11908696; doi:10.1371/journal.pgph.0003843)
Supplement: S1 Text — (DOCX) [file pgph.0003843.s001.docx]

**S1 Text: Study Protocol**

# Protocol for a scoping review of child public health indicators used in fragile, conflict-affected and vulnerable settings

## Rationale

*“It is a simple truth that in times of crisis, children suffer most.”*^1^

Millions of children and young people are currently residing in humanitarian settings. Globally 1 in 4 children under 15 years of age reside in countries affected by humanitarian emergencies.^2^ Global Humanitarian Overview data showed that 149 million children were in need of assistance in 2022, compared to 123 million in the previous year and numbers are expected to increase due to conflict, climate change disasters, increasing food insecurity and ongoing forced displacement.^3^ Children are disproportionately affected by humanitarian crises. For example, children account for 30 per cent of the world’s population, but 41 per cent of all forcibly displaced people.^4^ Children and young people are particularly vulnerable to direct and indirect health harms associated with humanitarian crises with half of all deaths among children under five years occurring in humanitarian settings.^5^ Children experience high levels of morbidity and mortality and adverse impacts on their physical, psychological, social and emotional development and their growth. Exposure to adversities has a life-long impact on children’s health and wellbeing.^6^

The United Nations Convention on the Rights of the Child (UNCRC) defines states’ legal obligations to respect, protect and fulfil the rights of children – including a fundamental right to health.^7^ This International human rights law is applicable in armed conflict as well as peace time. The 2030 Agenda for Sustainable Development references children in most of its Sustainable Development Goals and stresses the role of preparedness and development programming to reduce needs, vulnerabilities and risks.^8^ However, despite their increased risk of health harms, population level data to inform understanding of the health needs of children in humanitarian settings is limited. This creates challenges to achieving commitments to address the health and wellbeing needs of children globally. Stakeholders cannot determine priority actions or objectively measure whether they are on track to realise their ambitions/goals without data. Effective humanitarian response requires access to timely and relevant data at crisis onset and the ability to collect, analyse and interpret data throughout the response. Data is essential for evidence-informed action. Relevant data collection can meaningfully inform policy and practice in humanitarian settings. A simplified linear illustration of the data to decision-making process is given in Figure 1. In reality the process is more complex and perhaps better illustrated by Figure 2 which shows a cycle of data-driven policy and decision-making.

#### Figure 1: From data to evidence-informed action


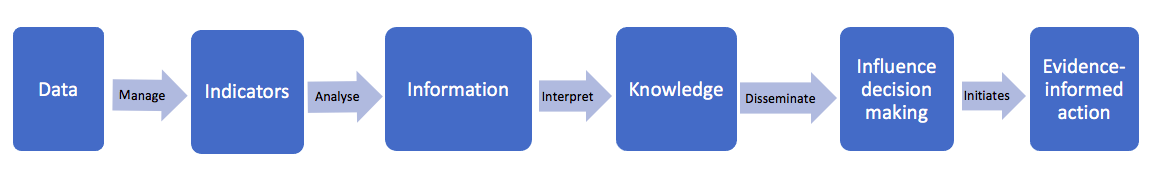


#### Figure 2: Cycle of data driven policy and decision making^9^


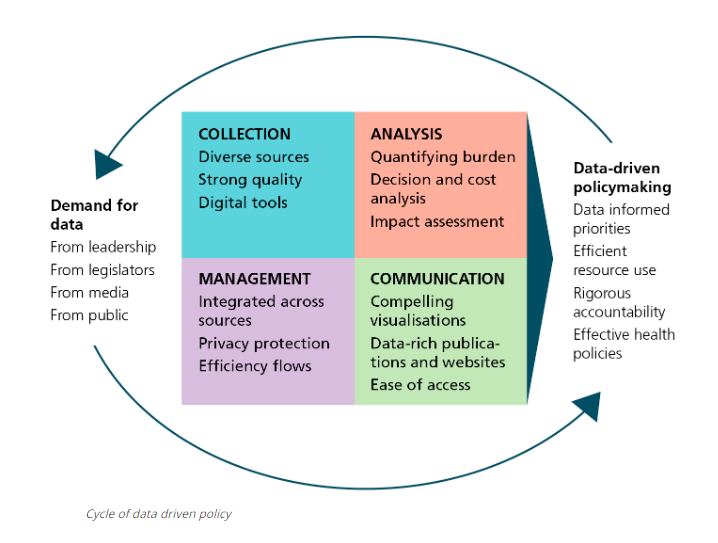


Current challenges to understanding the health needs of children in humanitarian settings are discussed below.

**Intermittent data collection**

Existing data is primarily collected by Multiple Indicator Cluster Surveys (MICS) and Demographic and Health Surveys (DHS). These intermittently conducted surveys provide cross-sectional data, which in many countries is outdated and represents the state of child population health over 10 years earlier.^10^ While MICS survey findings for some countries currently experiencing humanitarian crisis e.g. Afghanistan, Iraq, Yemen and Bangladesh, have been recently updated, data collection is not sufficient or regular enough to support crisis response, especially as people move or are displaced.

**Insufficient data on child public health**

Indicators used to inform decision-making for child population health improvement often represent a medicalised view of health, which risks neglecting the significant impact of the social (wider) determinants of health. Examples of indicators measuring the social determinants of child health include those related to: childhood poverty; educational attainment/school attendance; homelessness; access to safe water; latrine and soap access; access to food; and household/community violence. The impact of adversity on child development, physical and mental health outcomes, and mortality is well documented in high income stable settings, yet child exposure to adversity is not routinely measured in fragile, conflict-affected or vulnerable settings.^11^ Decision-makers need to understand all factors that influence child health, and to what extent, if they are to effectively prioritise where to invest resources to improve the health of the child population.

Child public health indicators are more likely to provide decision-makers with the ‘bigger picture’. They enable identification of intervention opportunities upstream of health problems occurring and recognition of actions required in parallel to healthcare interventions. This could ensure medium and long-term improvement in child population health. For example, provision of treatment for pneumonia in children is important, but access to data that identifies low child vaccination rates could prompt decision-makers to invest in childhood vaccination and reduce the risk of infection (re)occurring. Similarly, data demonstrating association of severe acute malnutrition (SAM) with a population’s poor access to clean water and sanitation could lead to investment in water, sanitation and hygiene (WASH) interventions to address the determinants for SAM, rather than isolated end-stage problem-oriented response of treating acute malnutrition and discharging patients back to conditions that place them at risk for relapse. Integrated analysis of a population’s child health indicators and general public health indicators is necessary; this requires a shift from silo working of humanitarian programmes.

**Lack of consensus on the definition of a child**

The United Nations Convention on the Rights of the Child (UNCRC) defines a child as ‘every human being below the age of eighteen years unless under the law applicable to the child, majority is attained earlier.’^7^ Thus, even this internationally accepted definition is ambiguous as countries have different definitions of ‘a child’ stated in various legislations for specific purpose. Furthermore, in some cultures the definition of a child is related to biological sciences, with a child considered to be a person between birth and puberty. In other cultures, adulthood may be perceived as reached when a child has undergone a rite of passage. Without a globally agreed standard objective universal definition of a child for use in the context of reporting child health indicators, it will not be possible to ensure comprehensive data collection for direct comparisons on the state of child health populations between settings and over time.

**Lack of standardised age disaggregation**

Age disaggregation of health data is not consistent. The Global Burden of Disease (GBD) study is the most comprehensive worldwide observational epidemiological study but the published prevalence, incidence and mortality data is not currently disaggregated by age. This is true of many global, country level and local data-sets. Age disaggregation does present challenges, particularly in FCV settings. Exact age and birth dates are not routinely collected – or in some cases are unknown or falsely reported; older children and adolescents may be omitted from household surveys; and individual level data collection is often paper based and processes used for aggregating individual data at local level to national level are inefficient.^12^ However, it is important to disaggregate data by age as different age groups have different health needs, risk factors and outcomes. Age disaggregation generates an understanding of the burden of disease within different age groups. This is necessary to inform actions to improve child population health. For example, neonates and infants are at high risk of adverse health outcomes from infectious diseases, environmental exposures and injuries are prevalent causes of mortality amongst 1-9 year olds, and adolescents experience greater morbidity from mental, sexual and reproductive health problems than younger children. Age disaggregation of data on risky health behaviours and their outcomes are particularly important.^12^ For example, examination of age disaggregated data for smoking, substance misuse, and high risk sexual behaviour is necessary to inform policies and ensure prevention programmes target groups at highest risk.

Data on children are often aggregated differently across indicators and programme/project-based priorities, making it difficult to compare or pool data. This, in turn, impedes pooling, analysis and interpretation of data across indicators. There is a strong case for standardised age disaggregation in order to improve the usability and comparability of data across and within countries and regions, and over time.^12^ By collecting and analysing data by standardised age groups, stakeholders can better understand the unique health needs of different strata of the child population; identify meaningful correlations among various factors, effectively target prevention interventions to the age groups that would deliver greatest population health benefit; prioritise resources for age groups that would benefit most; and implement responsive relevant actions to appropriate age groups. Furthermore, it would inform quantitative programme evaluations, to measure causal inference, and determine best practices.^12^

**Limitations in public health infrastructure in humanitarian settings**

In crisis contexts, where public health infrastructure may be disrupted or overwhelmed, lack of baseline data on child public health hampers appropriate and effective interventions due to:

1. Limited awareness or understanding of the distribution of health risks and needs of the population of children and vulnerable subgroups (e.g. infants, adolescents) - this may hamper appropriateness and effectiveness of humanitarian programming for children

2. Inability to monitor changes in children's health risks and needs as the crisis evolves

3. Inability to meaningfully measure quality and impact of response efforts for children.

Availability of baseline data could inform emergency preparedness, resilience and response planning, guide acute humanitarian response and facilitate measurement of its impact over time. Children's immediate and long-term health and development outcomes at any stage in childhood are heavily determined by their health up to that point. This is much more pronounced for children compared with adults, because children are growing and developing both physically as well as emotionally and socially, and they are adapting to their circumstances. Their behaviour and risks change as they grow, develop, and adapt. The ability to track what happens with health and development in a population over time is hugely important to understand the longer-term development and health outcomes, which can give insight into fundamental trends in child health risks and outcomes. Child public health data collection and analysis should continue during and post crisis as data-informed decision making is important for both humanitarian and development programmes.

A recommended set of relevant core child public health indicators that is comprised of realistically obtainable data elements in humanitarian settings is needed to align response to children’s needs, ensure accountable action, track progress over time, and enable a coordinated and consistent approach to measuring child public health between agencies working in humanitarian settings.

## Proposed study

A scoping review exploring the existing indicators currently used or recommended to measure the state of child health from a public health perspective in fragile, conflict-affected and vulnerable (FCV) settings will be conducted. The review will help to characterise the way child public health is currently measured in different contexts and identify any adaptations to indicators for populations in FCV settings. Understanding the current indicator landscape, including the similarities and differences in how child public health is measured by different agencies within and across settings, will provide insight into prioritised areas and the potential for harmonisation of measurements and pooling of data. The study will shed light on limitations and knowledge gaps in child public health in FCV settings, and inform further research and discourse about priority core child public health indicators for routine collection in FCV settings. Such a resource would be beneficial for ministries of health, humanitarian organisations, donors, service providers, and policy makers at local, regional, national and global levels.

The decision to expand the review scope to include indicators in FCV settings rather than just humanitarian settings is due to the aforementioned need for baseline data on child public health to inform humanitarian response. Humanitarian crises are more likely to occur or have recently occurred in FCV settings. Therefore, identification of which indicators are currently used or recommended for use in FCV settings would generate an understanding of information that may be available at the onset of a humanitarian crisis as well as an understanding of changes in data collection during and beyond the crisis period. FCV settings were selected rather than low and middle-income countries as FCV settings are more likely to experience the same challenges with data collection and analysis as humanitarian settings.

## Definitions and concepts

**Fragile, conflict-affected and vulnerable (FCV)** settings is a term used by WHO to describe a range of situations including humanitarian crises, protracted emergencies and armed conflicts.^13^ In general, FCV settings experience disruption of routine health service organization and delivery systems, increased health needs, complex resourcing landscapes, and vulnerability to further public health crises. The World Bank uses the term ‘fragile’ to define settings facing particularly severe development challenges such as weak institutional capacity, poor governance or political instability. For the purpose of this scoping review, we have adopted a definition proffered by RELIEF International: settings where multiple overlapping and interconnected challenges result in an accumulation of risks that cause a collapse of services, leading to local populations being unable to access the basic necessities they need to survive and thrive.^14^ It includes settings at high risk of experiencing an acute humanitarian crisis, settings experiencing humanitarian crises, and settings recovering from humanitarian crises.

A **humanitarian setting** has been defined by WHO as one in which ‘an event (e.g. armed conflict, natural disaster, epidemic, famine) or a series of events has resulted in a critical threat to health, safety, security and well-being of a community or other large group of people; he coping capacity of the affected community is overwhelmed and external assistance is required’.^15^

**Health** is defined by the World Health Organization (WHO) as “a state of complete physical, mental, and social well-being and not merely the absence of disease or infirmity”’.^16^

As previously discussed, the definition of a **child** is not universally agreed, but for the purpose of this scoping review the **child population** is defined as all human beings up to the age of 18 years. In this review the term **younger children** refers to children up to 10 years of age and **adolescents** refers to children aged 10-18 – the age 10 cut-off being consistent with WHO definition of an adolescent which is 10-19 years.^17^

**Indicators** are tools used to measure and describe a system or situation. In the context of FCV settings and humanitarian response indicators can be categorised as situation indicators, response monitoring indicators, and impact indicators.^18^ **Situation indicators** can describe what the current situation is (baseline indicators) or describe what is required in crisis-affected areas (needs indicators). Response monitoring indicators include input, output, and outcome indicators. **Input indicators** demonstrate the financial and human resources provided for the response, **output indicators** are usually a quantitative summary of response activity**, outcome indicators** are a measure of the change that has occurred as a result of the response**. Impact indicators** measure the short and medium to long term results of an intervention. This scoping review is interested in: situation baseline and needs indicators; response outcome indicators; and impact indicators. Input and output indicators are more likely to be programme/organisation specific and are less useful in generating understanding of the child public health landscape.

**Public Health** is defined as "the science and art of preventing disease, prolonging life and promoting health through the organized efforts and informed choices of society, organizations, public and private, communities and individuals”.^19^

**Child public health action** has been defined as: (1) action at policy, organization and local level to improve the overall health of children and young people; (2) action at policy, organization and local level to reduce inequalities in the health of children and young people; and (3) advocating for the rights of children and young people.^20^ Child public health interventions seek to both prevent and mitigate health harms at a child population level and across sectors.

For the purpose of this review ‘**child public health indicators**’ are defined as summary measures that capture information on different attributes of children’s health status, the social determinants of child health, healthcare in relation to the child population and health behaviours that influence child health. Analysis of child public health indicators should provide an overview of the determinants of child health in a given population and the existing or potential health risks and needs. Child public health indicators should generate strategic health information that can be meaningfully used by decision makers to inform actions and programmes to improve the health of the child population, increase life expectancy and address health inequalities.

The authors have identified four child public health indicator areas for exploration within this review (Figure 3). They are intentionally broad and have been selected with the logic that they would capture information related to the overlapping and interdependent public health practice domains of Health Protection, Health Improvement and Health Service Delivery.

- ***Health status*** refers to states of good health and normal development, as well as morbidity and mortality related to physical and mental health, disease outbreaks and immunisation, nutrition, disability, psychomotor and social development, and social wellbeing in the child population.
- ***Healthcare*** encompasses both access to and quality of health service provision to children. Access barriers may be financial (direct or indirect), geographical, cultural, related to language or literacy, or associated with individual or institutional discrimination. WHO defines quality healthcare as services which are effective, safe, people-centred, timely, equitable, integrated and efficient.^21^ Quality healthcare for children in FCV settings includes access to trauma-informed, clinically trained child health professionals who have the equipment, medications and appropriate context-specific guidance/protocols to address the physical, emotional and psychological needs of the child population in a safe environment.
- The ***social determinants of health*** are defined by WHO as “the conditions in which people are born, grow, work, live, and age, and the wider set of forces and systems shaping the conditions of daily life.”^22^ The social determinants of health in fragile, conflict affected and vulnerable settings are broad but key areas relevant to child health have been identified based on humanitarian response clusters.^23^ These include: child safety and protection; education and school attendance; food security; water, sanitation and hygiene (WASH); and shelter. Weakening of familial or communal protective mechanisms is a recognised risk factor for increased vulnerability to adverse health outcomes in children in FCV settings.^24^ Children in FCV settings are at increased risk of experiencing death of a parent, separation from their caregivers, family and/or communities, and having a caregiver who is physically or mentally ill or disabled; therefore any indicators covering this area should also be included.
- **Health behaviours** are intentional or unintentional actions taken by individuals that affect health or mortality either negatively or positively.^25^ Examples include smoking, substance use, diet, physical activity, sleep, risky sexual activities, healthcare seeking behaviours, and compliance with medical advice/treatments. Health behaviours may be influenced by the social determinants of health and by the social norms people are exposed to, both of which may be adversely impacted in times of humanitarian crises. Health behaviours of children themselves, their caregivers and community members can all impact child population health.

The authors recognise the impact that maternal health has on a child’s health and wellbeing, however inclusion of indicators covering this is beyond the scope of this review. Indicators included in the report will focus on the living child. Indicators associated with maternal health will only be included in contexts where the pregnant female is a child (<19 years of age).

#### Figure 3: Child public health indicator areas


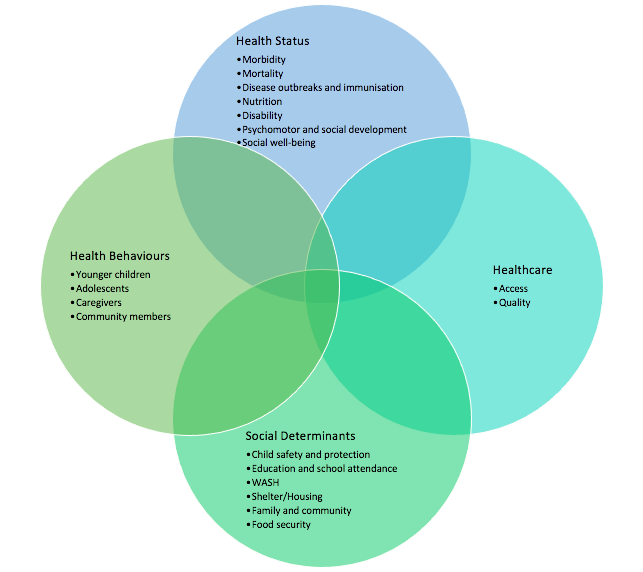


The same indicator may be relevant to more than one area e.g. Childhood vaccination coverage may: (1) provide information on health status in terms of risk of communicable disease outbreaks in the population; (2) provide information on vaccination service coverage which is linked to healthcare; and (3) may offer insight into vaccination uptake rates which is associated with health behaviours.

## Review objectives and research questions

There are two elements to the review:

1. To map child public health indicators **recommended** for use in FCV settings

2. To identify the child public health indicators that are actually **used** by different agencies and organisations working in FCV settings.

The objectives of the review are: to determine what child public health indicators exist for FCV settings; define what they measure; highlight discrepancies between recommended indicators and indicators used in practice; identify variations of similar indicators that are used in practice; and uncover measurement gaps.

The review will be guided by the following research questions:

- What child public health indicators have been recommended for or are currently used in FCV settings?
  - How is the indicator defined? This includes the definition of a child as well as data elements.
  - Which organisation/institution is recommending/using the indicator?
  - What adaptions have been made for FCV settings?
  - What age disaggregation is used, if any?
  - Is the data disaggregated by gender?
  - Is the indicator used for routine measurement, surveillance or surveys?
  - Is it a situation (baseline or crisis), response monitoring (outcome) or impact indicator?
  - Has the indicator undergone a quality check/review process before being used/recommended for use?

## Search strategy

1. Identifying recommended indicators

The reviewers will identify recommended indicators to measure child public health in FCV settings through a review of databases (e.g. PubMed, EMBASE, DuckDuckGo), relevant organisation webpages (e.g. WHO, UNICEF, UNHCR, OCHA (ReliefWeb), Global Health Cluster, USAID, FCDO, humanitarian agencies) and expert consultation. Published academic articles and grey literature will be reviewed. All literature identified for review must be freely available to the reviewers, presented in English language, and published in or after the year 2013. Possible search terms that could be used to identify relevant literature are given in Table 1. Where available, truncation and wildcard functions for keywords and index term functions (e.g. MeSH, Emtree) will be used within databases to minimise risk of missing relevant articles. Reference lists of relevant documents will be scanned to identify other relevant papers. The search strategy used for each database will be recorded for replicability.

#### Table 1: Possible search terms for concepts of interest

| **Concept** | **Possible search terms** |
| --- | --- |
| FCV setting | ‘Fragile, conflict-affected and vulnerable’, FCV, fragile setting, humanitarian, natural disaster, disaster, emergency, crisis, war, conflict, famine, refugee camp, displaced person camp, low-income country |
| Child | Neonate, newborn, infant, child, children, adolescent, young person/people, youth, teenager, teen |
| Public Health | Public health, health, wellbeing, health care, social determinants, wider determinants, health behaviour, lifestyle, |
| Indicator | Health indicator, indicator, data element |

If not publicly available, reviewers will reach out to the following organisations to request information on the child public health indicators they recommend for routine collection in FCV settings:

- World Health Organisation (WHO) – Departments responsible for: Quality of Care; Maternal, Newborn, Child and Adolescent Health and Ageing; Social Determinants of Health; Health Emergencies, Preparedness and Response; Nutrition and Food Safety; WASH, the Child Health Accountability Tracking technical advisory group (CHAT), Mother and Newborn Information for Tracking Outcomes and Results Advisory Group (MoNITOR) and the Global Action for Measurement of Adolescent Health (GAMA)
- United Nations Children’s Fund (UNICEF)
- United Nations High Commissioner for Refugees (UNHCR)
- International Organization for Migration (IOM)
- United Nations Office for Coordination of Humanitarian Affairs (OCHA)
- Global Health Cluster
- Save The Children International (SCI)
- Médecins Du Monde (MdM)
- Médecins Sans Frontières (MSF)
- International Committee of the Red Cross (ICRC)
- International Rescue Committee (IRC)
- United States Agency for International Development (USAID)
- UK Foreign, Commonwealth and Development Office (FCDO)
- Bill & Melinda Gates Foundation

Reports/papers identified by the search strategy will then be screened to determine whether they meet all of the following inclusion criteria for further review:

- Recommends indicators that inform understanding of child public health in FCV settings
- Includes a definition of the recommended child public health indicators

Reports/papers which focus on reporting of child public health outcomes rather than recommending child public health indicators for use in FCV settings will be excluded.

2. Identifying indicators actively used in FCV settings

Reviewers will reach out to the following organisations to request information on the child public health indicators they currently routinely collect in the FCV settings they work in:

- World Health Organisation (WHO) – Departments responsible for: Quality of Care; Maternal, Newborn, Child and Adolescent Health and Ageing; Social Determinants of Health; Health Emergencies, Preparedness and Response; Nutrition and Food Safety; and WASH
- United Nations Children’s Fund (UNICEF)
- United Nations High Commissioner for Refugees (UNHCR)
- International Organization for Migration (IOM)
- United Nations Office for Coordination of Humanitarian Affairs (OCHA)
- Save The Children International (SCI)
- Médecins Du Monde (MdM)
- Médecins Sans Frontières (MSF)
- International Committee of the Red Cross (ICRC)
- International Rescue Committee (IRC)

## Indicator inclusion criteria

The reviewers will then examine the indicator lists obtained through application of the search strategy and select indicators for inclusion in the review using the following criteria:

- Be child-focused. The indicators must directly pertain to children 0-18 years or a disaggregated child age group in this bracket.
- Be used or recommended for use/used in in FCV settings.
- Be relevant to at least one of the defined child public health domains – health status, health service access and quality, social determinants of health, health behaviours.
- Contain data and good metadata (title, definition, and description of how the indicator is calculated).
- Be a situation, outcome response, or impact indictor. Input, process or output indicators will be excluded.

## Compilation and analysis of indicators

For each indicator relevant metadata will be extracted in to a spreadsheet with the following headings:

- Report/database title
- Organisation(s)/Institution(s)/Author(s)
- Relevant expert’s name, role and contact email address
- Reference
- Indicator name
- Indicator definition and components, including data element(s)
- Age disaggregation is used, if any
- Gender disaggregations, if any
- Any other disaggregations
- Indicator type: situation, outcome, impact
- Adaptations for humanitarian settings, if any
- In use or recommended
- Additional notes

All indicators will be coded according to thematic focus areas

- Child public health area: health status, health services, social determinants, and health behaviour)
- Indicator quality will be coded based on degree of interpretation required to measure the indicator
- Free text will be used to code for additional characteristics that are not listed in the codebook
- The drafted codebook will be piloted by three researchers on a sample of the dataset and refined accordingly.
- See codebook for code definitions and examples

Analysis will focus on characterisation of the numbers and types of indicators, similarities and differences between indicators, similarities and differences in disaggregation, identification of thematic topics with more developed or heavier focus, gaps in measurement, and the degree of interpretation required to measure each indicator. Similarities and differences between recommended, required, and routinely measured indicators will be explored.

## Recommended follow-on work

- Carry out a Delphi study with expert representatives working in the main child public health areas and agencies working in FCV settings to achieve a consensus view on which standardised core child public health indicators should be prioritised for use in FCV settings to inform effective data driven decision making? This should include a quality assessment (e.g. flexible/ measurable/ feasible/ valid/ timely/ replicable/ sustainable/ relevant/ important/ comprehensible?) of the situation, outcome and impact indicators identified in the scoping review and consideration of gap analysis findings.
- Identification and publication of a short list of core recommended child public health indicators for use in FCV settings.

## References

1. UNICEF. Humanitarian Action for Children [Internet]. 2021. Available from: https://www.unicef.org/media/88416/file/HAC-2021-overview.pdf

2. WHO Regional Office for the Mediterranean. Child and adolescent health in humanitarian settings: operational guide [Internet]. 2021 [cited 2023 Jan 18]. Available from: https://applications.emro.who.int/docs/9789290228172-eng.pdf

3. OCHA. Global Humanitarian Overview 2023. [Internet]. 2023 [cited 2023 Jan 18]. Available from: https://humanitarianaction.info/?gclid=CjwKCAiAh9qdBhAOEiwAvxIokwKp0uGC68j0umcwP8vV0OrLDABRHBUJBKhKpj5FrVooMTaBE2uehBoCCeUQAvD_BwE

4. UNHCR. Global Trends Report 2021 [Internet]. 2021 [cited 2023 Jan 18]. Available from: https://www.unhcr.org/uk/publications/brochures/62a9d1494/global-trends-report-2021.html

5. WHO. Children in humanitarian settings. [Internet]. [cited 2023 Jan 18]. Available from: https://www.who.int/teams/maternal-newborn-child-adolescent-health-and-ageing/child-health/children-in-humanitarian-settings

6. NCSL. Adverse Childhood Experiences [Internet]. 2022 [cited 2023 Jan 18]. Available from: https://www.ncsl.org/health/adverse-childhood-experiences

7. UN. The United Nations Convention on the Rights of the Child. 1989 [cited 2023 Jan 18]; Available from: https://www.unicef.org.uk/wp-content/uploads/2016/08/unicef-convention-rights-child-uncrc.pdf

8. UNDP. Sustainable Development Goals [Internet]. 2022 [cited 2023 Jan 18]. Available from: https://www.undp.org/sustainable-development-goals?utm_source=EN&utm_medium=GSR&utm_content=US_UNDP_PaidSearch_Brand_English&utm_campaign=CENTRAL&c_src=CENTRAL&c_src2=GSR&gclid=CjwKCAiAzp6eBhByEiwA_gGq5KAQfewTy4330kt8OAyj-bS0zjboc2SKrTcnRwvhA9OlW-VVC0AMCx

9. Global Health Data Methods. Data to drive public health policy [Internet]. 2019 [cited 2023 Jan 31]. Available from: https://globalhealthdata.org/data-for-public-health-policy/

10. UNICEF. MICS Surveys [Internet]. 2023 [cited 2023 Jan 18]. Available from: https://mics.unicef.org/surveys

11. Oh DL, Jerman P, Silvério Marques S, Koita K, Purewal Boparai SK, Burke Harris N, et al. Systematic review of pediatric health outcomes associated with childhood adversity. BMC Pediatr [Internet]. 2018 Feb 23 [cited 2023 Jan 18];18(1):1–19. Available from: https://bmcpediatr.biomedcentral.com/articles/10.1186/s12887-018-1037-7

12. Diaz T, Strong KL, Cao B, Guthold R, Moran AC, Moller AB, et al. A call for standardised age-disaggregated health data. Lancet Heal Longev [Internet]. 2021 Jul 1 [cited 2023 Jan 18];2(7):e436–43. Available from: http://www.thelancet.com/article/S266675682100115X/fulltext

13. WHO. Quality of care in fragile, conflict-affected and vulnerable settings [Internet]. [cited 2023 Jan 18]. Available from: https://www.who.int/teams/integrated-health-services/quality-health-services/quality-of-care-in-fragile-conflict-affected-and-vulnerable-settings

14. Relief International. Fragile Settings [Internet]. [cited 2023 Jan 18]. Available from: https://www.ri.org/fragile-settings/

15. WHO. Inter-Agency Field Manual on Reproductive Health in Humanitarian Settings. Inter-agency F Man Reprod Heal Humanit Settings [Internet]. 2010 [cited 2023 Jan 18];1–222. Available from: https://www.ncbi.nlm.nih.gov/books/NBK305149/

16. WHO. Constitution of the World Health Organization [Internet]. [cited 2023 Jan 18]. Available from: https://www.who.int/about/governance/constitution

17. WHO. Adolescent health [Internet]. [cited 2023 Jan 18]. Available from: https://www.who.int/health-topics/adolescent-health#tab=tab_1

18. Strachan AL. Indicators. In: Humanitarianism: Keywords [Internet]. Brill; 2020 [cited 2023 Jan 18]. p. 112–4. Available from: https://brill.com/display/book/edcoll/9789004431140/BP000052.xml

19. WHO Regional Office for Europe. Strengthening Public Health Capacity and Services in Europe. 2011 [cited 2023 Jan 18]; Available from: http://www.euro.who.int/pubrequest

20. Cresswell T. What is child public health? Curr Paediatr [Internet]. 2004 Dec 1 [cited 2023 Jan 18];14(7):612–8. Available from: http://www.paediatricsandchildhealthjournal.co.uk/article/S0957583904001150/fulltext

21. WHO. Quality of care [Internet]. [cited 2023 Jan 18]. Available from: https://www.who.int/health-topics/quality-of-care#tab=tab_1

22. WHO. Social determinants of health [Internet]. [cited 2023 Jan 18]. Available from: https://www.who.int/health-topics/social-determinants-of-health#tab=tab_1

23. OCHA. What is the Cluster Approach? [Internet]. [cited 2023 Jan 18]. Available from: https://www.humanitarianresponse.info/en/coordination/clusters/what-cluster-approach

24. Wise PH, Shiel A, Southard N, Bendavid E, Welsh J, Stedman S, et al. The political and security dimensions of the humanitarian health response to violent conflict. Lancet [Internet]. 2021 Feb 6 [cited 2023 Jan 18];397(10273):511–21. Available from: http://www.thelancet.com/article/S0140673621001306/fulltext

25. Short SE, Mollborn S. Social Determinants and Health Behaviors: Conceptual Frames and Empirical Advances. Curr Opin Psychol [Internet]. 2015 Oct 1 [cited 2023 Jan 18];5:78. Available from: /pmc/articles/PMC4511598/

**Database searches**

**Embase search 24 March 2024**

Embase <1974 to 2023 March 23>

1 ('fragile conflict affected and vulnerable').mp. [mp=title, abstract, heading word, drug trade name, original title, device manufacturer, drug manufacturer, device trade name, keyword heading word, floating subheading word, candidate term word] 9

2 ((humanitarian or refugee* or conflict*) adj6 (crisis or crises or disaster* or relief or intervention* or aid)).mp. [mp=title, abstract, heading word, drug trade name, original title, device manufacturer, drug manufacturer, device trade name, keyword heading word, floating subheading word, candidate term word] 6494

3 humanitarian aid/ or humanitarian crisis/ 470

4 natural disaster/ 4252

5 war/ 32040

6 ((fragile or vulnerable or conflict*) adj5 (setting* or context* or countr*)).mp. [mp=title, abstract, heading word, drug trade name, original title, device manufacturer, drug manufacturer, device trade name, keyword heading word, floating subheading word, candidate term word] 5595

7 ((refugee* or displaced person* or displaced people or migrant*) adj5 (housing or camp* or shelter* or accomodation*)).mp. [mp=title, abstract, heading word, drug trade name, original title, device manufacturer, drug manufacturer, device trade name, keyword heading word, floating subheading word, candidate term word] 2795

8 refugee camp/ 1078

9 natural disaster*.mp. [mp=title, abstract, heading word, drug trade name, original title, device manufacturer, drug manufacturer, device trade name, keyword heading word, floating subheading word, candidate term word] 8431

10 1 or 2 or 3 or 4 or 5 or 6 or 7 or 8 or 9 52740

11 child*.mp. [mp=title, abstract, heading word, drug trade name, original title, device manufacturer, drug manufacturer, device trade name, keyword heading word, floating subheading word, candidate term word] 3145969

12 juvenile/ or exp adolescent/ or exp child/ 3929478

13 (newborn* or neonate* or infant*).mp. [mp=title, abstract, heading word, drug trade name, original title, device manufacturer, drug manufacturer, device trade name, keyword heading word, floating subheading word, candidate term word] 1438166

14 infant/ 689915

15 11 or 12 or 13 or 14 4694707

16 public health.mp. [mp=title, abstract, heading word, drug trade name, original title, device manufacturer, drug manufacturer, device trade name, keyword heading word, floating subheading word, candidate term word] 576186

17 exp public health/ 232382

18 exp public health surveillance/ 678

19 health*.mp. [mp=title, abstract, heading word, drug trade name, original title, device manufacturer, drug manufacturer, device trade name, keyword heading word, floating subheading word, candidate term word] 6455935

20 ('social determinants of health' or 'wider determinants of health').mp. [mp=title, abstract, heading word, drug trade name, original title, device manufacturer, drug manufacturer, device trade name, keyword heading word, floating subheading word, candidate term word] 23131

21 (morbidity or mortality or nutrition or disabilit*).mp. [mp=title, abstract, heading word, drug trade name, original title, device manufacturer, drug manufacturer, device trade name, keyword heading word, floating subheading word, candidate term word] 2910010

22 (disease* or infection* or vaccin* or immunisation*).mp. [mp=title, abstract, heading word, drug trade name, original title, device manufacturer, drug manufacturer, device trade name, keyword heading word, floating subheading word, candidate term word] 12463246

23 (water or sanitation or sanitar* or hygien* or WASH).mp. [mp=title, abstract, heading word, drug trade name, original title, device manufacturer, drug manufacturer, device trade name, keyword heading word, floating subheading word, candidate term word] 1540551

24 ((child* adj2 (protection or welfare or safety)) or safeguard*).mp. [mp=title, abstract, heading word, drug trade name, original title, device manufacturer, drug manufacturer, device trade name, keyword heading word, floating subheading word, candidate term word] 47385

25 (education* or school*).mp. [mp=title, abstract, heading word, drug trade name, original title, device manufacturer, drug manufacturer, device trade name, keyword heading word, floating subheading word, candidate term word] 2179157

26 (house* or housing or shelter*).mp. [mp=title, abstract, heading word, drug trade name, original title, device manufacturer, drug manufacturer, device trade name, keyword heading word, floating subheading word, candidate term word] 381453

27 (wellbeing or well being or wellness).mp. [mp=title, abstract, heading word, drug trade name, original title, device manufacturer, drug manufacturer, device trade name, keyword heading word, floating subheading word, candidate term word] 221722

28 ('child development' or 'psychomotor development').mp. [mp=title, abstract, heading word, drug trade name, original title, device manufacturer, drug manufacturer, device trade name, keyword heading word, floating subheading word, candidate term word] 62613

29 ((food or nutrition*) adj3 secur*).mp. [mp=title, abstract, heading word, drug trade name, original title, device manufacturer, drug manufacturer, device trade name, keyword heading word, floating subheading word, candidate term word] 17084

30 "social determinants of health"/ 18170

31 exp health care facility/ 1902990

32 exp health service/ 6689903

33 sexual health/ 20882

34 exp health status/ 303735

35 exp health status indicator/ 40792

36 exp health/ 870654

37 health impact assessment/ 7629

38 exp health care access/ 98630

39 exp health care system/ 660220

40 preventive health service/ 30559

41 exp health literacy/ 18669

42 health statistics/ 2888

43 exp health care/ 6312803

44 exp health behavior/ 487276

45 exp community mental health service/ 867

46 exp school health service/ 20914

47 exp health data/ 575305

48 exp health care utilization/ 92254

49 exp health promotion/ 114228

50 exp health education/ 371390

51 community mental health/ 4077

52 health equity/ 9286

53 exp health care need/ 38728

54 exp health care quality/ 4091459

55 exp health practitioner/ 60977

56 exp health hazard/ 665457

57 exp morbidity/ 430523

58 exp mortality/ 1355308

59 exp nutrition/ 2613680

60 exp disability/ 188276

61 mental disease/ 269893

62 diseases/ or exp "general aspects of disease"/ or exp mental disease/ or exp physical disease/ 25572800

63 exp infection/ 4029615

64 exp immunization/ 363630

65 exp water/ 561796

66 exp sanitation/ 540837

67 exp hygiene/ 90558

68 exp child welfare/ 18566

69 exp education/ 1649672

70 exp housing/ or exp housing quality/ 31562

71 exp wellbeing/ 119982

72 exp child development/ 49449

73 psychomotor development/ 5992

74 food security/ 8187

75 16 or 17 or 18 or 19 or 20 or 21 or 22 or 23 or 24 or 25 or 26 or 27 or 28 or 29 or 30 or 31 or 32 or 33 or 34 or 35 or 36 or 37 or 38 or 39 or 40 or 41 or 42 or 43 or 44 or 45 or 46 or 47 or 48 or 49 or 50 or 51 or 52 or 53 or 54 or 55 or 56 or 57 or 58 or 59 or 60 or 61 or 62 or 63 or 64 or 65 or 66 or 67 or 68 or 69 or 70 or 71 or 72 or 73 or 74 30765113

76 indicator*.mp. [mp=title, abstract, heading word, drug trade name, original title, device manufacturer, drug manufacturer, device trade name, keyword heading word, floating subheading word, candidate term word] 488799

77 exp health status indicator/ 40792

78 exp performance indicator/ 3144

79 clinical indicator/ 5757

80 76 or 77 or 78 or 79 524966

81 humanitarian.mp. [mp=title, abstract, heading word, drug trade name, original title, device manufacturer, drug manufacturer, device trade name, keyword heading word, floating subheading word, candidate term word] 7842

82 10 or 81 57038

83 15 and 75 and 80 and 82 408

84 limit 83 to yr="2012 -Current" 285

85 limit 84 to english language 281

**Medline search 24 March 2024**

Ovid MEDLINE(R) ALL <1946 to March 23, 2023>

1 ('fragile conflict affected and vulnerable').mp. [mp=title, book title, abstract, original title, name of substance word, subject heading word, floating sub-heading word, keyword heading word, organism supplementary concept word, protocol supplementary concept word, rare disease supplementary concept word, unique identifier, synonyms, population supplementary concept word, anatomy supplementary concept word] 8

2 ((humanitarian or refugee* or conflict*) adj6 (crisis or crises or disaster* or relief or intervention* or aid)).mp. [mp=title, book title, abstract, original title, name of substance word, subject heading word, floating sub-heading word, keyword heading word, organism supplementary concept word, protocol supplementary concept word, rare disease supplementary concept word, unique identifier, synonyms, population supplementary concept word, anatomy supplementary concept word] 5755

3 exp Relief Work/ 6005

4 exp Natural Disasters/ 25045

5 exp "Warfare and Armed Conflicts"/ 48145

6 ((fragile or vulnerable) adj5 (setting* or context* or countr*)).mp. [mp=title, book title, abstract, original title, name of substance word, subject heading word, floating sub-heading word, keyword heading word, organism supplementary concept word, protocol supplementary concept word, rare disease supplementary concept word, unique identifier, synonyms, population supplementary concept word, anatomy supplementary concept word] 1952

7 ((refugee* or displaced person* or displaced people or migrant*) adj5 (housing or camp* or shelter* or accomodation*)).mp. [mp=title, book title, abstract, original title, name of substance word, subject heading word, floating sub-heading word, keyword heading word, organism supplementary concept word, protocol supplementary concept word, rare disease supplementary concept word, unique identifier, synonyms, population supplementary concept word, anatomy supplementary concept word] 2270

8 Refugee Camps/ 287

9 natural disaster*.mp. [mp=title, book title, abstract, original title, name of substance word, subject heading word, floating sub-heading word, keyword heading word, organism supplementary concept word, protocol supplementary concept word, rare disease supplementary concept word, unique identifier, synonyms, population supplementary concept word, anatomy supplementary concept word] 5960

10 1 or 2 or 3 or 4 or 5 or 6 or 7 or 8 or 9 89285

11 child*.mp. [mp=title, book title, abstract, original title, name of substance word, subject heading word, floating sub-heading word, keyword heading word, organism supplementary concept word, protocol supplementary concept word, rare disease supplementary concept word, unique identifier, synonyms, population supplementary concept word, anatomy supplementary concept word] 2728767

12 exp Child/ 2133450

13 Adolescent/ 2206093

14 (newborn* or neonate* or infant*).mp. [mp=title, book title, abstract, original title, name of substance word, subject heading word, floating sub-heading word, keyword heading word, organism supplementary concept word, protocol supplementary concept word, rare disease supplementary concept word, unique identifier, synonyms, population supplementary concept word, anatomy supplementary concept word] 1548319

15 exp Infant/ 1243531

16 11 or 12 or 13 or 14 or 15 4580124

17 public health.mp. [mp=title, book title, abstract, original title, name of substance word, subject heading word, floating sub-heading word, keyword heading word, organism supplementary concept word, protocol supplementary concept word, rare disease supplementary concept word, unique identifier, synonyms, population supplementary concept word, anatomy supplementary concept word] 419670

18 exp Public Health/ 9135825

19 Public Health Surveillance/ 5214

20 health*.mp. [mp=title, book title, abstract, original title, name of substance word, subject heading word, floating sub-heading word, keyword heading word, organism supplementary concept word, protocol supplementary concept word, rare disease supplementary concept word, unique identifier, synonyms, population supplementary concept word, anatomy supplementary concept word] 4555216

21 ('social determinants of health' or 'wider determinants of health').mp. [mp=title, book title, abstract, original title, name of substance word, subject heading word, floating sub-heading word, keyword heading word, organism supplementary concept word, protocol supplementary concept word, rare disease supplementary concept word, unique identifier, synonyms, population supplementary concept word, anatomy supplementary concept word] 13992

22 exp Health Facilities/ 887056

23 exp Health Services/ 2413693

24 exp Health/ 431239

25 Health Impact Assessment/ 930

26 exp Health Status/ 431831

27 exp Health Status Indicators/ 341168

28 exp School Health Services/ 24386

29 exp Health Services Accessibility/ 133848

30 "Delivery of Health Care"/ 114726

31 exp Preventive Health Services/ 677667

32 exp Emergency Medical Services/ 167081

33 exp Primary Health Care/ 189182

34 exp Health Behavior/ 358638

35 Community Mental Health Services/ 19018

36 exp Dental Care/ 34645

37 exp Rural Health Services/ 14020

38 exp Population Health/ 40673

39 secondary care/ or tertiary healthcare/ 2656

40 exp Health Promotion/ 84744

41 Nutritional Status/ 53302

42 exp Health Education/ 261448

43 Health Equity/ 3373

44 exp "Quality of Health Care"/ 8048505

45 (morbidity or mortality or nutrition* or disabilit*).mp. [mp=title, book title, abstract, original title, name of substance word, subject heading word, floating sub-heading word, keyword heading word, organism supplementary concept word, protocol supplementary concept word, rare disease supplementary concept word, unique identifier, synonyms, population supplementary concept word, anatomy supplementary concept word] 2275240

46 (disease* or infection* or vaccin* or immunisation*).mp. [mp=title, book title, abstract, original title, name of substance word, subject heading word, floating sub-heading word, keyword heading word, organism supplementary concept word, protocol supplementary concept word, rare disease supplementary concept word, unique identifier, synonyms, population supplementary concept word, anatomy supplementary concept word] 8909011

47 (water or sanitation or sanitar* or hygien* or WASH).mp. [mp=title, book title, abstract, original title, name of substance word, subject heading word, floating sub-heading word, keyword heading word, organism supplementary concept word, protocol supplementary concept word, rare disease supplementary concept word, unique identifier, synonyms, population supplementary concept word, anatomy supplementary concept word] 1290779

48 ((child* adj2 (protection or welfare or safety)) or safeguard*).mp. [mp=title, book title, abstract, original title, name of substance word, subject heading word, floating sub-heading word, keyword heading word, organism supplementary concept word, protocol supplementary concept word, rare disease supplementary concept word, unique identifier, synonyms, population supplementary concept word, anatomy supplementary concept word] 44689

49 (education* or school*).mp. [mp=title, book title, abstract, original title, name of substance word, subject heading word, floating sub-heading word, keyword heading word, organism supplementary concept word, protocol supplementary concept word, rare disease supplementary concept word, unique identifier, synonyms, population supplementary concept word, anatomy supplementary concept word] 1391030

50 (house* or housing or shelter*).mp. [mp=title, book title, abstract, original title, name of substance word, subject heading word, floating sub-heading word, keyword heading word, organism supplementary concept word, protocol supplementary concept word, rare disease supplementary concept word, unique identifier, synonyms, population supplementary concept word, anatomy supplementary concept word] 298862

51 (wellbeing or well being or wellness).mp. [mp=title, book title, abstract, original title, name of substance word, subject heading word, floating sub-heading word, keyword heading word, organism supplementary concept word, protocol supplementary concept word, rare disease supplementary concept word, unique identifier, synonyms, population supplementary concept word, anatomy supplementary concept word] 147605

52 ('child development' or 'psychomotor development').mp. [mp=title, book title, abstract, original title, name of substance word, subject heading word, floating sub-heading word, keyword heading word, organism supplementary concept word, protocol supplementary concept word, rare disease supplementary concept word, unique identifier, synonyms, population supplementary concept word, anatomy supplementary concept word] 65315

53 ((food or nutrition*) adj3 secur*).mp. [mp=title, book title, abstract, original title, name of substance word, subject heading word, floating sub-heading word, keyword heading word, organism supplementary concept word, protocol supplementary concept word, rare disease supplementary concept word, unique identifier, synonyms, population supplementary concept word, anatomy supplementary concept word] 14407

54 exp Morbidity/ 643743

55 exp Mortality/ 422180

56 nutrition assessment/ 17588

57 exp Nutrition Disorders/ 420868

58 exp Disease/ 190346

59 exp Infections/ 3039322

60 exp Immunization/ 206642

61 exp Water/ 213430

62 sanitary engineering/ or sanitation/ 10173

63 exp Hygiene/ 45371

64 exp Child Welfare/ 32035

65 exp Education/ 894016

66 exp Housing/ 36489

67 exp "Quality of Life"/ 262211

68 exp Child Development/ 66157

69 exp food insecurity/ or exp food security/ 1857

70 17 or 18 or 19 or 20 or 21 or 22 or 23 or 24 or 25 or 26 or 27 or 28 or 29 or 30 or 31 or 32 or 33 or 34 or 35 or 36 or 37 or 38 or 39 or 40 or 41 or 42 or 43 or 44 or 45 or 46 or 47 or 48 or 49 or 50 or 51 or 52 or 53 or 54 or 55 or 56 or 57 or 58 or 59 or 60 or 61 or 62 or 63 or 64 or 65 or 66 or 67 or 68 or 69 19475198

71 indicator*.mp. [mp=title, book title, abstract, original title, name of substance word, subject heading word, floating sub-heading word, keyword heading word, organism supplementary concept word, protocol supplementary concept word, rare disease supplementary concept word, unique identifier, synonyms, population supplementary concept word, anatomy supplementary concept word] 442163

72 exp Quality Indicators, Health Care/ 24733

73 exp Health Status Indicators/ 341168

74 71 or 72 or 73 758445

75 humanitarian.mp. [mp=title, book title, abstract, original title, name of substance word, subject heading word, floating sub-heading word, keyword heading word, organism supplementary concept word, protocol supplementary concept word, rare disease supplementary concept word, unique identifier, synonyms, population supplementary concept word, anatomy supplementary concept word] 6904

76 10 or 75 92721

77 16 and 70 and 74 and 76 651

78 limit 77 to yr="2012 -Current" 389

79 limit 78 to english language 381
