# Supplementary material for: Child public health indicators for fragile, conflict-affected, and vulnerable settings: A scoping review
Source: PLOS Glob Public Health. 2025 Mar 14;5(3):e0003843. doi: 10.1371/journal.pgph.0003843 (PMC11908696; doi:10.1371/journal.pgph.0003843)
Supplement: S4 Text — (DOCX) [file pgph.0003843.s004.docx]

**S4: Sources of included indicators**

| Category of Indicator | Source |
| --- | --- |
| Routinely collected indicators | Save the Children International  Médecins Sans Frontières  International Committee of the Red Cross  International Rescue Committee  United Nations Children’s Fund |
| Required indicators | United States Agency for International Development Bureau for Humanitarian Affairs  European Civil Protection and Humanitarian Aid Operations |
| Recommended indicators for routine collection in FCV settings | Peer-reviewed publications  United Nations Office for the Coordination of Humanitarian Affairs  United Nations High Commission for Refugees  Sphere Handbook  Alliance for Child Protection in Humanitarian Action  Inter-Agency Working Group on Reproductive Health in Crises  Healthy Newborn Network  Inter-agency Network for Education in Emergencies  Interagency Gender Working Group |
