# Supplementary material for: Child public health indicators for fragile, conflict-affected, and vulnerable settings: A scoping review
Source: PLOS Glob Public Health. 2025 Mar 14;5(3):e0003843. doi: 10.1371/journal.pgph.0003843 (PMC11908696; doi:10.1371/journal.pgph.0003843)
Supplement: Attachment — Submitted filename: Response to Reviewers.docx [file pgph.0003843.s010.docx]

**Response to reviewers**

**Editor comments**

1. Please define and distinguish between 'humanitarian', 'fragile', 'conflict-affected' and 'vulnerable' settings or explain why these terms are used interchangeably.

Thank you for this comment, it is very important that we are clear about the meaning and use of the terms. The terms “humanitarian” and “fragile, conflict-affected and vulnerable” are not used interchangeably in the paper.

Fragile, conflict-affected and vulnerable (FCV) settings is defined in Table 1. We have refined the FCV definition, included an additional definition of humanitarian settings, differentiated between the two in the definitions table, and added text to the methods to explain the use of the terms. The term “humanitarian“ implies that external actors are required to respond to the needs of the affected populations. FCV settings do not necessarily require external assistance to support population needs. FCV also includes pre-crisis and protracted crises. When considering data collection, this becomes important because access and infrastructure in more stable FCV settings may make it easier to reliably collect child public health data.

2. Table 1 cites a broad WHO definition for FCV, but does not explain whether this is an objective or subjective classification or how it was applied in search and screening criteria. Is there a list of countries or sub-national contexts that are considered FCV at any given time? Were articles included only if authors used these exact terms to describe settings? Or if settings of interest met certain criteria? Further explanation will both enhance the replicability of the review, and guide readers in interpretation of recommended indicators and measurement methods.
   We have clarified the definition of FCV settings in Table 1 to indicate that it is subjective. We searched the literature for indicators that were routinely used, recommended or required for collection in any setting that falls under the broadest definition of FCV settings. We have adapted the text in the methods section and Table 3 to clarify the inclusion criteria for records retrieved through database searches.

**Review Comments to the Author**

**Reviewer #1**: The author addresses a relevant topic in public health highlighting the gaps in children's public health data in fragile, conflict-affected, and vulnerable settings (FCV). They provided in their introduction, a comprehensive overview of the challenges faced by children during FCV and the need for more standardized children's public health indicators. However, it could benefit from clearer structure and concision. They presented many relevant findings determining trends and gaps in the current system. Providing more actionable recommendations would enhance that work. The article is comprehensive overall, but would benefit of more concision and organization.

Thank you for your feedback and the comments which have helped us to strengthen the manuscript.

Major recommendations
Abstract
1. Conclusion
- Findings Implications: In the Conclusions section, the first sentence seemed to be weak. Consider highlighting the implications of your findings. For example, discuss the feasibility of measuring child public health outcomes in FCV settings despite challenges, and its relevance. The practical implications of the excessive number and poor definition of indicators recommended by technical experts.

We have adjusted the text to strengthen the summary of key findings and their implications as well as recommendations (pages 2-3 lines 45-54). The key finding from this study is that child public health outcomes are already being routinely measured. As such, the rationale frequently used by humanitarian actors for not collecting outcomes – that it is just too difficult to do - is not valid.

2. - Recommendations: The recommendation may be more concise. In addition to the consensus, consider emphasizing needs for standardization, prioritization and addressing the gaps identified in the study. An final sentence about the finality of that recommendation would be good like : to improve monitoring and evaluation in these settings or improve child healh during FCV or....
We have adapted the recommendation in the abstract (page 3 lines 49-54).

Background
3. While the introduction covers a broad spectrum of challenges and contexts, ensure that the narrative consistently emphasizes the public health aspects relevant to children in FCV settings.The introduction highlighted the problem of humanitarian data limitation and most importantly the mismatch between publicly available data and tons of internal data collected by NGOs. The paragraph after explained the lack of standardization of those data. The authors may consider presenting those arguments in more integrated and concise ways to emphasize their importance.

Thank you for this input. We agree, the introduction covered a number of important points in different parts, sometimes with a focus on more on issues relating to iNGOs, the aid industry, and on the larger population. We have revised the text to retain focus on child public health in FCV settings.

4. There is a lack of connection between the idea expressed in the sentence from lines 66 to 68 and the one in lines 70-71. A better transition can be useful here. A better transition can also be done between sentences 84-86 and 88-89

We have revised the text to improve these transitions. (page 4 lines 73-78, and page 5 lines 98-100)

5. Make sure that each point stays connected back to the specific context of child public health in FCV settings and how the lack of data impacts public health responses. This ensures that the introduction remains focused on the study's relevance.

Thanks for this, we’ve revised the introduction text to further reinforce the focus on child public health in FCV settings, importance of data to inform public health responses, and impact of lack of data.

Methods
6. The Methods section contains important details but lacks clear organization and structure. It would benefit from separating different stages of the review process into distinct paragraphs or subsections, such as search strategy, screening process, inclusion criteria, and data analysis. It would be good as well to present a specific paragraph about the grey literature assessment. Were there pre-approved strategies to resolve disagreements among reviewers, if yes, specify it.

We have organised the text according to the components of the review, with a separate paragraph for the grey literature assessment. We have also specified the disagreement resolution strategy.

7. The authors specified that the DuckDuckGo search 153 was run on 14 March 2023. Is there a specific reason to provide a date only for that activity and not for the others? It would be better to know the general timeframe of the whole data collection process (from …. To …. ), instead of a single date for 1 activity.

The specific search dates for the database searches was also stated in the manuscript. We have now adapted the text to remove the specific search date for DuckDuckGo and added a more general phrase that all searches were run 14-24 March 2024.

8. Mention how inter-rater reliability was ensured during the screening and inclusion process. This could include details on pilot testing of the inclusion criteria and coding framework.

We have added text as requested.

Results
9. Adding the proportion to table 6, as it is done for table 7, will provide more insights.
We have added proportions to Table 6.

Discussion
10. The first paragraph of the discussion could synthesize better the scoping review's main findings.
We have adjusted the paragraph to strengthen it. Thee first paragraph of the discussion gives an overview of the study findings, and the main findings are discussed in detail in subsequent paragraphs. We have added an additional sentence to the first paragraph to bring attention to the most important finding – that child public health indicators, including outcome indicators, are routinely measured in spite of the contextual challenges.

11. Second paragraph of the discussion. More than 500 indicators are recommended. The authors can explain this more striking finding and its relevance. Do all those 500 indicators seem necessary? The authors mentioned they are not realistic for governments: specifying why would reinforce the statement (assess the 500 will require more funding, staffing, logistics, will not be sustainable … ? )
While the section discusses the large number of recommended indicators and feasibility issues, it could delve deeper into interpreting why these challenges exist. For instance, explore the underlying reasons for the discrepancy between recommended indicators and feasible measurements in FCV settings.
We have added further text to the paragraph to discuss the significance of the finding, some of the nuances relating to this, and the challenges posed by a large number of recommended indicators.

12. Consider strengthening the discussion of gaps in measuring early child development and disability by exploring the potential consequences of these gaps on program effectiveness and child health outcomes.
Thanks for this, we have added some text to address the above.

Conclusion and recommendation
13. The conclusion outlines key issues but lacks a clear pathway for addressing them. Instead of stating findings already explained in results and discussions, it could focus more on presenting a succinct summary of the implications of the findings for policy, practice, and future research and highlight actionable steps that stakeholders can take to address the identified challenges in measuring child public health in FCV settings.
We have made one specific recommendation for policy, research and practice, which has 3 components. We feel this is the first and most important action, and that the recommendation is succinct: “There is a need to establish consensus on priority child public health topics for measurement and a shortlist of high quality, appropriate and relevant measurements that governmental and nongovernmental actors can be reasonably expected to measure routinely in FCV settings, with proxy indicators for periods where routine measurement is hampered”. We have added additional text to recommend that selection and/or development of indicators should be done in a way that will be prioritise indicators that are most useful to guide decision-making by operational teams, recognising that the information needs for operational teams is dynamic, evolving throughout the crisis cycle.

There are a number of ways that consensus could be established. Assessment of the quality of indicators and prioritisation of core indicators could be done through a DELPHI, or perhaps a WHO-led process, either from the Cluster system or the Maternal, Newborn, Child, and Adolescent Health and Aging Department. We have intentionally not recommended specific actions because there are many constructive ways forward, and because this study identified the need for a bigger shift in thinking to recognise the child public health work contributions of sectors that are not traditionally seen as public health actors, especially the child protection and education sectors.

Minor recommendations
14. Ensure the abstract is concise and uses precise language.
We have adapted the text to make it more precise and address the reviewer’s suggestions

15. Line 46: instead of "proving this is possible" in the Conclusions section, you might say "demonstrating feasibility."
We have adjusted the wording.

16. Some sentences could be streamlined for clarity and conciseness. For example, sentences like "In the absence of routine child public health data, humanitarian actors delivering public health services in these settings often turn to survey data..." could be broken down into shorter, clearer segments for easier comprehension.
We have broken this down to shorter, more focused sentences.

17. Editing: conduct a thorough final reading for missing words or grammatical errors. For example, in Table 3. Correct impact indictors to impact indicators.

Apologies for the typo, we’ve corrected it and screened again for other typos.

**Reviewer #2:** A very useful review that presents a clear need for further standardization and alignment of child health indicators in FCV settings.
Thank you for this feedback and for the helpful comments and inputs, which have improved the paper.

The recommendations can be improved by considering the following:

1. Standardization may only be possible ‘within’ the data source type such as the following:

-Population-based household survey (from DHS down to local rapid household survey);
-Clinical information system such as paper logbooks or registers up to electronic registers or medical records (often called health management information system or routine health information system).
-Health facility assessment such as HERAMS, WHO Harmonized Health Facility, or rapid/local health facility assessments, which may include checks on availability of human resources and essential drugs/equipment, interviews with patients and health workers up to observations of care against clinical guidelines.
-Others (e.g., review of charts, clinical notes, other program records)
-As an example, the WHO quality of care indicator, “% sick children 2months – 5years assessed on IMCI Criteria and appropriately treated” is most appropriately assessed using observation by a independent clinical assessor when a guidelines-based electronic medical record is not available (e.g., WHO digital adaptation kit for child health in emergencies).

Thank you, this is an important point. We’ve added a paragraph to briefly address this.

2. Recommendations could provide a standard definition by data type. For example, coverage of measles immunization estimated by a household survey will be defined differently than coverage of measles immunization estimated through the clinical information system. One reason is that denominators will be defined differently or come from different information systems. Surveys provide their own denominator while clinical information systems rely on other sources such as OCHA for population in need or a separate enumeration or other projection. And often, the numerators are also obtained differently (e.g., mother’s report, home vaccination card, marker on child’s finger, or tally of vaccines distributed).

We’ve added a paragraph about standardisation based on the point above and addressed issues relating to definitions in that paragraph. We have made a more general recommendation that efforts to establish consensus on a short list of standardised child public health indicators must consider data sources that will be used for each indicator and provide clear definitions of the numerator and denominator.

3. A more general discussion of denominator issues in FCV settings, including how availability of denominators varies by data source, concerns about the accuracy of denominators used in clinical information systems even in more stable settings, etc. and its affect on standardization.

Thank you, this is a good point and a recurring discussion among the author group as we screened indicators. We’ve added a short discussion on challenges relating to denominators in FCV settings.
