# Supplementary material for: Child public health indicators for fragile, conflict-affected, and vulnerable settings: A scoping review
Source: PLOS Glob Public Health. 2025 Mar 14;5(3):e0003843. doi: 10.1371/journal.pgph.0003843 (PMC11908696; doi:10.1371/journal.pgph.0003843)
Supplement: Attachment — Submitted filename: Review_Child public health indicators for fragile.docx [file pgph.0003843.s007.docx]

**Review of “Child public health indicators for fragile, conflict-affected, and vulnerable settings: a scoping review**”

A very useful review that presents a clear need for further standardization and alignment of child health indicators in FCV settings.

The recommendations can be improved by considering the following:

- Standardization may only be possible ‘within’ the data source type such as the following:
  - Population-based household survey (from DHS down to local rapid household survey);
  - Clinical information system such as paper logbooks or registers up to electronic registers or medical records (often called health management information system or routine health information system).
  - Health facility assessment such as HERAMS, WHO Harmonized Health Facility, or rapid/local health facility assessments, which may include checks on availability of human resources and essential drugs/equipment, interviews with patients and health workers up to observations of care against clinical guidelines.
  - Others (e.g., review of charts, clinical notes, other program records)
  - As an example, the WHO quality of care indicator, “% sick children 2months – 5years assessed on IMCI Criteria and appropriately treated” is most appropriately assessed using observation by a independent clinical assessor when a guidelines-based electronic medical record is not available (e.g., WHO digital adaptation kit for child health in emergencies).
- Recommendations could provide a standard definition by data type. For example, coverage of measles immunization estimated by a household survey will be defined differently than coverage of measles immunization estimated through the clinical information system. One reason is that denominators will be defined differently or come from different information systems. Surveys provide their own denominator while clinical information systems rely on other sources such as OCHA for population in need or a separate enumeration or other projection. And often, the numerators are also obtained differently (e.g., mother’s report, home vaccination card, marker on child’s finger, or tally of vaccines distributed).
- A more general discussion of denominator issues in FCV settings, including how availability of denominators varies by data source, concerns about the accuracy of denominators used in clinical information systems even in more stable settings, etc. and its affect on standardization.
