# Supplementary material for: Child public health indicators for fragile, conflict-affected, and vulnerable settings: A scoping review
Source: PLOS Glob Public Health. 2025 Mar 14;5(3):e0003843. doi: 10.1371/journal.pgph.0003843 (PMC11908696; doi:10.1371/journal.pgph.0003843)
Supplement: Attachment — Submitted filename: Response to reviewers 2.docx [file pgph.0003843.s009.docx]

18 September 2024

Response to comments:

*1. Please send a completed 'Competing Interests' statement, including any COIs declared by your co-authors. If you have no competing interests to declare, please state "The authors have declared that no competing interests exist".*

Response: we state “The authors have declared that no competing interests exist"

*2. Please amend your detailed Financial Disclosure statement. This is published with the article. It must therefore be completed in full sentences and contain the exact wording you wish to be published.*

Response: This statement (“The authors received no specific funding for this work.”) has been added on line 281 (tracked changes version).

*3. Please upload all main figures as separate Figure files in .tif or .eps format only. For more information about how to convert and format your figure files please see our guidelines.*

Response: The image has been uploaded as a tif file.
